# Executive Summary – Guidelines for Mechanical Circulatory Support of the Brazilian Society of Cardiology

**DOI:** 10.5935/abc.20180126

**Published:** 2018-07

**Authors:** Silvia Moreira Ayub-Ferreira

**Affiliations:** Instituto do Coração do Hospital das Clínicas da Faculdade de Medicina da Universidade de São Paulo, São Paulo, SP – Brazil. Hospital Sírio-Libanês, São Paulo, SP – Brazil

**Keywords:** Heart Failure/complications, Heart Failure/therapy, Myocardial Ischemia/complications, Assisted Circulation/instrumentation, Contraindicators, Risk Assessment

## Evaluation of candidates for mechanical circulatory support devices

In advanced heart failure (HF), the Interagency Registry for Mechanically Assisted Circulatory Support (INTERMACS) proposed seven clinical profiles (and modifiers) for a convenient, easy classification of disease status, risk of implantation of mechanical circulatory support devices (MCSDs) and adequate time for intervention (Chart 1).^1^

**Chart 1:** Interagency Registry for Mechanically Assisted Circulatory Support (INTERMACS) profiles

Profile	Description	Hemodynamic status	Time frame for definitive intervention
1	Critical cardiogenic shock	Persistent hypotension despite the use of inotropes and intra-aortic balloon pumps, associated with organic dysfunction	Hours
2	Progressive decline, but inotrope dependent	Deterioration of renal and hepatic function, nutritional status and lactate levels, despite use of inotropes in optimized doses	Days
3	Stable but inotrope dependent	Clinical stability on continuous inotropic therapy, and history of failure to wean from it	Weeks - months
4	Frequent hospitalization	Signs of water retention, symptoms at rest and frequent admissions to emergency departments	Weeks - months
5	At home, exertion intolerant	Intolerant to activity, comfortable at rest despite water retention	Intervention emergency depends on nutritional status and organic dysfunction severity
6	Exertion limited	Moderate limitation to activity; absence of signs of hypervolemia	Intervention emergency depends on nutritional status and organic dysfunction severity
7	NYHA III	Hemodynamic stability and absence of hypervolemia	Intervention is not indicated

NYHA: New York Heart Association.

One of the main determinant factors for a successful MCSD implantation is patient eligibility. Correct selection of patients involves – (1) patients with advanced HF to which the risk of MCSD implantation surpasses mortality risk for current disease (making it a beneficial intervention); (2) patients with moderately advanced HF, i.e., implantation of MCSD would not increase patient’s morbidity and mortality due to increased complication rate; (3) no contraindications for MCSD implantation.^2,3^

Perioperative renal failure, pre-existing right HF, liver dysfunction, mechanical ventilation in the pre-operative period, low weight or overweight and reoperation have been related to worse clinical outcomes after MCSD implantation.^3-5^

The main scores for risk prediction in MCSD implantation are described in Chart 2.

**Chart 2:** Risk predictors for mechanical circulatory support device implantation

Risk score for destination therapy^6^	Risk score for bridge/destination therapy (HMII score)^7^	Pre-operative risk score^8^	Pre-operative risk score^9^
Risk of 90-day in-hospital mortality (pulsatile flow)	Ninety-day mortality (continuous flow)	Mortality risk after MCSD implantation (mean of 84 days)	Mortality risk after MCSD implantation (mean of 100 days)
Platelets < 148.000/µL OR: 7.7	Age (for 10 years) OR: 1.32	Urine flow < 30 mL/hour RR: 3.9	Respiratory failure /sepsis OR: 11,2
Albumin < 3.3 mg/dL OR: 5.7	Albumin OR: 0.49	CVP > 16 mmHg RR: 3.1	Right heart failure OR: 3.2
INR > 1,1 OR: 5.4	Creatinine OR: 2.1	Mechanical ventilation RR: 3	Age > 65 years OR: 3.01
Use of vasodilator OR: 5.2	INR OR: 3.11	Prothrombin time > 16 seconds RR: 2.4	Postcardiotomy acute ventricular failure OR: 1.8
Pulmonary artery medium pressure < 25 mmHg OR: 4.1	Center volume < 15 implants OR: 2.24	Reoperation RR: 1.8	Acute myocardial infarction OR: 1.7
ALT > 45 U/mL OR: 2.6		Leucocytes > 15.000 RR: 1.1	
Hematocrit < 34% OR: 3,0		Temperature > 101.5 F RR: 0	
BUN > 51 U/dL OR: 2.9			
Intravenous inotropic support OR: 2.9			

HMII: HeartmateII; OR: odds ratio; RR: relative risk; CVP: central venous pressure; INR: international normalized ratio; ALT: alanine transaminase; BUN: Blood Urea Nitrogen. MCSD: mechanical circulatory support device

## Echocardiography

Evaluation of patients candidates for MCDS should include a transthoracic echocardiogram (TEE) complemented by a transesophageal echocardiography (TEE).

The effects of MCDS on right ventricular function depend on the balance between the benefits of decompression of the left chambers (reduction of the left ventricular afterload) and greater volumetric load to the right atrium (RA; increase of the right ventricular preload). Decompression of left chambers also cause changes in the geometry of the right chambers, such as leftward shift of interatrial (IAS) and interventricular septum (IVS), structural changes of tricuspid annulus, which can aggravate a pre-existing tricuspid insufficiency (TI) and right ventricular overload.^10^

Considering that right ventricular cardiac output determines left ventricular preload, a significant reduction in right ventricular function results in decreased output by the MCSD. It is estimated that approximately 30% of patients with left ventricular assist device develop limiting right ventricular dysfunction. For these reasons, a careful evaluation of right ventricular function is mandatory before MCDS implantation. In the presence of moderate-to-severe dysfunction, the requirement of a permanent biventricular support cannot be ruled out.^11^

In the assessment of right ventricular function before MCSD implantation, it is recommended the measurement of the right ventricle, as well as a semiquantitative assessment of right ventricular longitudinal and radial contractility combined with quantitative parameters, including fractional area change (FAC; FAC < 20% are associated with increased risk of right ventricular dysfunction after MCSD implantation),^12^ tricuspid annular plane systolic excursion (TAPSE) determined by M mode, peak systolic velocity of lateral tricuspid ring, measured by tissue Doppler (s’), and right ventricular performance index.^13,14^

### Predictors of right ventricular dysfunction before mechanical circulatory support device implantation

Right ventricular dysfunction is multifactorial and includes an increase in preload, ventricular ischemia and mechanical interdependence of ventricular geometry. It is one of the most severe complications of left ventricular assist device, observed in up to 30% of cases and associated with a six-fold increase in morbidity and mortality (increased risk in up to 67%).^11,15^

Risk factors and the main risk score for right ventricular dysfunction after MCSD implantation are described in Charts 3 and 4.

**Chart 3:** Risk factors for right ventricular dysfunction after mechanical circulatory support device implantation (MCSD)^16^

Indication of MCDS	Destination therapy
Sex	Female
Pre-implantation support	Intra-aortic balloon pump and vasopressor requirement
Organic dysfunctions	Respiratory: invasive ventilatory support
	Hepatic: ALT ≥ 80 UI/L. bilirubin > 2.0 mg/dL
	Renal: serum creatinine ≥ 2.3 g/dLHistory of kidney replacement therapy
	Nutritional: albumin ≤ 3.0 g/dL
	Coagulation: platelets < 120,000
	Others: increased BNP. PCR. Procalcitonin
Right ventricular dysfunction	Right ventricular diastolic diameter > 35 mm. FAC < 30%. Right atrium > 50 mm
Hemodynamic measures	CVP ≥ 15 mmHg or CVP/PCP ≥ 0.63. right ventricular work index ≤ 300 mmHg mL/m^2^; low pulmonary artery pressures, low cardiac index or increased pulmonary vascular resistance
Others	Non-ischemic cardiomyopathy, reoperation, important TI, history of PTE

ALT: alanine transaminase; BNP: brain natriuretic peptide; CRP: C-reactive protein; FAC: fractional area change; CVP: central venous pressure; PCP pulmonary capillary pressure; TI: tricuspid insufficiency; PTE: pulmonary thromboembolism

**Chart 4:** Main risk scores for right ventricular failure after left ventricular mechanical circulatory support device implantation

Score	Variables	Prediction
University of Michigan, RV Failure Risk Score, Matthews et al.^17^	Vasopressor requirement: 4 points TGP ≥ 80 IU/L: 2 points Bilirubin ≥ 2.0 mg/dL: 2.5 points Creatinine ≥ 2.3 mg/dL or hemodialysis: 3 points	Likelihood of right ventricular failure • ≥ 5.5 points: 7.6 • 4.0-5.0 points: 2.8 • ≤ 3.0 points: 0.49
Kormos et al.^18^	Pre-operative predictors for early left ventricular dysfunction: CVP/PCP > 0.63 Ventilatory support BUN > 39 mg/dL	One-year survival: • Absent right ventricular dysfunction: 78% • Early right ventricular dysfunction: 59% (p**< 0.001)
University of Pennsylvania, RV Failure Risk Score, Fitzpatrick et al.^19^	Cardiac index ≤ 2.2 L/min/m^2^: 18 points SVRI ≤ 0.25 mmHg-L/m^2^: 18 points Important right ventricular dysfunction: 17 points Serum creatinine ≥ 1.9 mg/dL: 17 points Previous cardiac surgery: 16 points Systolic arterial pressure ≤ 96 mmHg: 13 points	< 30: 96%, isolated left ventricular assist device ≥ 65 points: 11%, isolated left ventricular assist device
CRITT score^20^	CVP > 15 mmHg: 1 point Severe right ventricular dysfunction: 1 point Pre-operative mechanical ventilation: 1 point Important tricuspid insufficiency: 1 point Tachycardia (> 100 bpm) = 1 point	1-2 points: low risk for right ventricular dysfunction 2-3 points: moderate risk for right ventricular dysfunction 4-5 points: high risk for right ventricular dysfunction

ALT: alanine transaminase; CVP: central venous pressure; PCP pulmonary capillary pressure; BUN: Blood Urea Nitrogen; SVRI: systemic vascular resistance index

Implantation of a MCSD in the left ventricle should be performed with caution in patients with important right ventricular dilation, moderate-to-severe tricuspid insufficiency, tricuspid valve annulus > 45 mm and CVP > 15 mmHg. By this means, hemodynamic variables directly reflect a preload or afterload increase and right ventricular contractility reductions, whereas venous congestion and organ hypoperfusion, consequence of right ventricular dysfunction, indicate hepatic and renal dysfunctions^15,21^

Positive hemodynamic indicators of adequate right ventricular function that might reduce the risk of post-MCSD implantation dysfunction are: CVP ≤ 8 mmHg; PCP ≤ 18 mmHg; CVP/PCP ≤ 0,66; pulmonary vascular resistance (PVR) < 2 wood units and right ventricular work index ≥ 400 mL/m^2^.

## Temporary devices

### Selection of strategies for temporary mechanical circulatory support devices

Temporary MCSD can be used for hemodynamic and clinical stability restoration, aiming at improvement of cardiac function and transplantation. Three strategies (which may be overlapped) can be defined:

1. **Bridge to decision:** should be considered in severely ill patients, who requires immediate hemodynamic support due to high risk of cardiac failure. It may occur in different situations – lack of neurological recovery, multiple organ failure, hemodynamic stabilization and requirement of other devices – in which the final strategy of therapy cannot be established during device implantation (e.g. after cardiorespiratory arrest).^22^
2. **Bridge to recovery:** situation in which support device is removed for ventricular function recovery, such as ventricular dysfunction following acute myocardial infarction, Takotsubo cardiomyopathy and myocarditis.^23^
3. **Bridge to transplantation:** situations in which the patient is in progressive severity and heart transplantation cannot be performed in a short term. Support devices may provide hemodynamic support and clinical stability until transplantation is performed.

### Types of temporary mechanical circulatory support devices 

Main characteristics of temporary MCSDs available in Brazil are described in Chart 5.^24^

**Chart 5:** Temporary mechanical circulatory support devices available in Brazil

Characteristics	Intra-aortic balloon	ECMO	TandemHeart™	Impella 2.5® Impella CP® Impella 5.0®	CentriMag®	EXCOR®
Mechanism	Pneumatic	Centrifugal	Centrifugal	Axial	Centrifugal	Pulsatile
Access	Percutaneous	Percutaneous / thoracotomy	Percutaneous	Percutaneous Percutaneous Dissection	Thoracotomy	Thoracotomy
Cannulation	7-9 F	18-21 F Inflow 15-22 F Outflow	21 F Inflow 15-17 F Outflow	12 F 14 F 21 F	24-34 F	27-48 F Inflow 36-48 F Outflow
Insertion technique	Descending aorta via femoral artery	Percutaneous: - Inflow: right atrium via femoral or jugular vein - Outflow: descending aorta via femoral artery Thoracotomy: - Inflow: right atrium - Outflow: pulmonary artery (left mechanical circulatory assist device) or ascending aorta (biventricular assist device)	Inflow: left atrium via femoral vein and transseptal puncture Outflow: femoral artery	Insertion into left ventricle via femoral artery	ACM-E: - Inflow: left ventricle (via left atrium or apex of left ventricle) - Outflow: ascending aorta ACM-D:- Inflow: right atrium - Outflow: pulmonary artery	ACM-E: - Inflow: left ventricle (apex of left ventricle) - Outflow: ascending aorta ACM-D: - Inflow: right atrium - Outflow: pulmonary artery
Hemodynamic support	0.5 L/min	> 4.5 L/min	4 L/min	2.5 L/min 3.7 L/min 5.0 L/min	Up to 8-10 L/min	Up to 8 L/min

ECMO: Extracorporeal membrane oxygenation

#### Indications and contraindications

Although temporary MCSDs are primarily indicated for patients INTERMACS levels 1 and 2, INTERMACS level 3 patients, dependent of high doses of inotropes or at high risk of hemodynamic instability may also be considered eligible.

Contraindications for temporary MCDS include limiting clinical situations that require individualized approach and involvement of other professionals (e.g. oncologist for establishment of cancer prognosis).

#### Intra-aortic balloon pump (IABP)

The mechanism of action of the IABP is aortic counterpulsation, which increases diastolic pressure at aortic root, promoting an increase in coronary perfusion, afterload reduction, and consequently an increment in cardiac output by 15%.

Although IABP is still used in the clinical practice especially in younger patients with less severe cardiogenic shock, the efficacy of the method should be carefully evaluated based on improvement of objective parameters of tissue microperfusion. Lack of improvement of these variables in a short time period (hours) justifies the selection of more invasive devices.

**Table:** Recommendations for intra-aortic balloon pump implantation

Recommendation	Class	Evidence level
Post-AMI cardiogenic shock	IIa	B
Post-AMI mechanical complication with cardiogenic shock	IIa	C
Refractory angina after standard therapy for acute coronary syndrome	IIa	C
Cardiogenic shock in ischemic / non-ischemic chronic cardiomyopathy	IIa	C
Intervention support for patients at high cardiac risk	IIb	C

AMI: acute myocardial infarction

### Percutaneous circulatory devices

#### Definition and benefits

Percutaneous circulatory devices enable active support without requiring a synchronism with the cardiac cycle. The main benefits are maintenance of tissue perfusion, improvement of coronary perfusion, and reduction of myocardial oxygen consumption, filling pressures and ventricular wall stress, providing a circulatory support in cardiogenic shock.^25,26^

**Table:** Recommendations for percutaneous circulatory support device implantation

Recommendation	Class	Evidence level
Post-AMI cardiogenic shock	IIa	C
Support for interventions in patients at high cardiac risk	IIb	C

AMI: acute myocardial infarction

### Types of percutaneous circulatory devices

#### Impella^®^

Impella device is composed of a continuous axial flow pump, that aspirates blood directly from the left ventricle and directs it to the aorta (works in series with left ventricle). It allows the flow of 2.5 L/min (Impella® 2.5), 4.0 L/min (Impella® CP) or 5.0 L/min (Impella® 5.0). The model currently available in Brazil is Impella® CP.^24,27^

#### TandemHeart™

TandemHeart™ system is composed of a centrifugal extracorporeal pump, a femoral cannula, a transseptal cannula and a control console. It pumps blood from the left atrium through a transseptal cannula to the ileo-femoral arterial system. Both TandemHeart™ and the left ventricle work in parallel and contribute to aortic blood flow.^24,27^

### Extracorporeal membrane oxygenation

#### Definition, types and benefits

Extracorporeal membrane oxygenation (ECMO) is an invasive temporary mechanical support that provides partial or total cardiopulmonary support for patients with cardiogenic shock and/or acute respiratory insufficiency. There are two types of ECMO – venoarterial and venovenous. With quick installation technology, ECMO promotes rapid reversal of circulatory failure and/or anoxia.

**Table:** Recommendations for extracorporeal membrane oxygenation implantation

Recommendation	Class	Level of evidence
Bridge to decision or recovery	I	C
Bridge to transplantation	IIa	C

### Paracorporeal circulatory support

#### Definition, types and benefits

Paracorporeal circulatory support devices are surgically implanted pumps that promote hemodynamic support in individuals with refractory cardiogenic shock with high mortality risk.

A CentriMag® is a continuous flow, magnetically levitated centrifugal blood pump. It provides up to 10 L/minute of blood flow and low shear stress, promoting low thrombogenicity, moderate anticoagulation levels and minimum hemolysis during support.^24^

Berlin Heart EXCOR® is a pulsatile-flow pump that provides up to 8 L/min of blood flow, with batteries connected to a transport system, allowing an up to ten hours of patient’s mobility.

**Table:** Recommendations for implantation of paracorporeal circulatory pumps

Recommendation	Class	Level of evidence
Bridge to decision or recovery	IIa	C
Bridge to transplantation	IIa	C

Other conventional centrifugal pumps may be used with the same purpose.

### Long term devices

#### Types of long-term mechanical circulatory support devices

**Figure 1:**
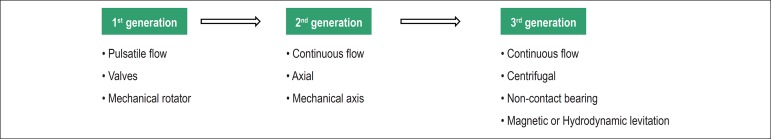
Progress of long-term mechanical circulatory support devices.

Due to technological progress, advances in long-term MCSD models have occurred during the last years, regarding pumping system and flow type, enabling its reduction in size, greater efficiency and lower complication rates (Figure 1).

The long-term MCSDs available in Brazil are described in Chart 6.

**Chart 6:** Long-term mechanical circulatory support devices available in Brazil

Name	Company	Type of pump	Type of support	Presence of bearing	Anvisa Approval
HeartMate II^®^	Thoratec	Axial flow	Left	Yes	Yes
INCOR^®^	Berlin Heart	Axial flow	Left	No (electromagnetic levitation)	Yes
HeartWare^®^	HeartWare	Centrifugal flow	Left	No (electromagnetic levitation)	Yes

Anvisa: Agência Nacional de Vigilância Sanitária (The Brazilian Health Regulatory Agency); NA: not applicable

#### Indications and contraindications

In making decision process for long-term MCSDs, some important factors should be considered. In case of bridge to transplantation, transplant waiting time should be taken into account; for waiting time shorter than 30 days, there would be a low benefit-cost ratio. Also, the use of these devices in INTERMACS level 2 patients may have unfavorable results.

**Table:** Recommendations for long-term mechanical circulatory support devices as bridge to transplant

Recommendation	Class	Level of evidence
Systolic heart failure - INTERMACS levels 2 and 3	Class IIa	C
Systolic heart failure - INTERMACS level 4	Class IIb	C
Systolic heart failure -INTERMACS levels 1, 5, 6 and 7	Class III	C

**Table:** Recommendations for long-term mechanical circulatory support devices as destination therapy

Recommendation	Class	Level of evidence
Systolic heart failure - INTERMACS 3	Class IIa	B
Systolic heart failure - INTERMACS 2		C
Systolic heart failure - INTERMACS 4	Class IIb	C
Systolic heart failure - INTERMACS 1, 5, 6 e 7	Class III	C

**Table:** Recommendations for long-term mechanical circulatory support devices as bridge to decision

Recommendation	Class	Level of evidence
Systolic heart failure - INTERMACS 2 and 3	Class IIa	C
Systolic heart failure - INTERMACS 4	Class IIb	C
Systolic heart failure - INTERMACS 1, 5, 6 and 7	Class III	C

Patients eligible for MCSD should be evaluated for the presence of factors that may contraindicate or negatively influence patients’ survival after transplant. Main contraindications are listed in Chart 7.

**Chart 7:** Contraindications for long-term mechanical circulatory support devices

**Absolute**	Coumarin intolerance
	Absence of trained caregivers
	Severe psychiatric disorders or nonadherence to the staff instructions
	Severe motor deficit or cognitive deficit related after stroke
	Neoplastic disease with unfavorable prognosis
	Vascular malformation of the small bowel that predisposes to bleeding
	Severe pulmonary obstructive disease
	Severe hepatic dysfunction
	Active infection
	Hematologic changes (platelets < 50,000 mm^3^ and thrombophilia)
**Relative**	Moderate-to-severe right ventricular dysfunction
	Dialytic therapy for renal failure
	Difficult-to-control diabetes
	Partial motor deficit after stroke
	Severe malnutrition
	Significative peripheral artery disease

#### Strategy for selection of long-term MCSDs

1. **Bridge to decision:** long-term MCSDs may be indicated for patients with clinical conditions that contraindicate heart transplantation, but if modified, patients may become eligible for transplant (for example: pulmonary hypertension and curable cancers).
2. **Bridge to transplant:** Situations in which MCSDs may provide hemodynamic support and clinical stability until heart transplant, in patients with progressive severity and when a short-term transplant is not possible.
3. **Destination therapy:** Situations in which MCSDs may provide hemodynamic support and clinical stability in patients with refractory heart failure with contraindication for cardiac transplant, promoting higher survival and better quality of life as compared with clinical treatment with drugs.

#### Optimization and management of right ventricular function

Right ventricular failure is still one of the main factors that affect patients’ survival after MCSD implantation.^28^ Its diagnostic criteria are – signs and symptoms for persistent right ventricular dysfunction; CVP > 18 mmHg with cardiac index < 2,0 L/min.m^2^ in the absence of ventricular arrhythmias or pneumothorax; requirement of ventricular support devices; or requirement for inhaled nitric oxide or inotropic therapy for more than one week after device implantation.^29^

Implantation of a MCSD increases cardiac output and consequently causes an increment in venous return to the right ventricle. To counteract this preload increase, right ventricular compliance should improve with reduction of its afterload (decrease in left ventricular filling pressure and pulmonary arterial pressure). However, leftward shift of IVS may occur in case of excessive left ventricular emptying.^29^

**Figure 2:**
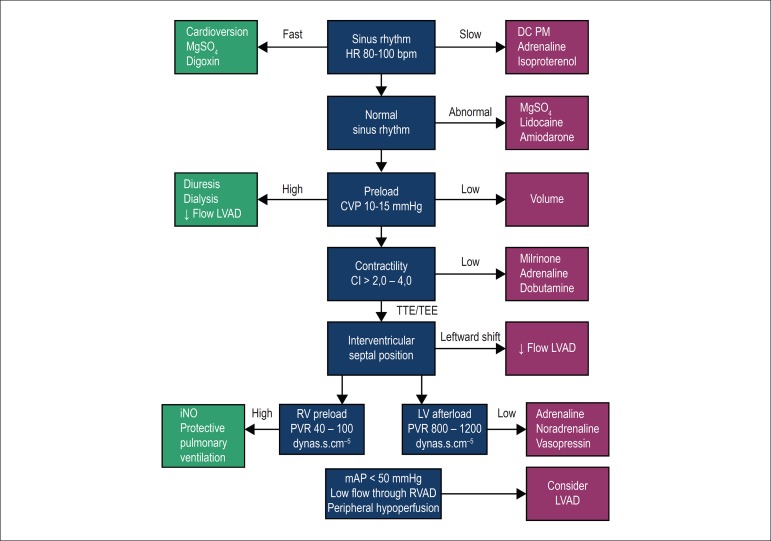
Optimization and management of right ventricular function. MgSO_4_: magnesium sulfate; HR: heart rate; DC PM: dual-chamber pacemaker with right atrial and ventricular stimulation and sensitivity; LVAD: Left ventricular assist device; CVP: central venous pressure; CI: cardiac index; TTE: transthoracic echocardiogram; TEE: transesophageal echocardiography; RV: right ventricular; PVR: pulmonary vascular resistance; LV: left ventricular; SVR: systemic vascular resistance; RVAD: right ventricular assist device; mAP: mean arterial pressure.

In addition to its contractility, optimization of right ventricular preload and afterload is crucial to prevent right ventricular failure in the perioperative period. CVP and systolic pulmonary pressure should be maintained lower than 16 mmHg and 65 mmHg, respectively. For maintenance of coronary perfusion, use of inotropes that cause pulmonary vasodilation (milrinone or dobutamine) and maintain adequate systemic pressure (adrenaline) is recommended. In addition, the use of specific pulmonary vasodilators, such as nitric oxide should be considered (Figure 2).^30^

#### Complications after long-term MCSD implantation

The main complications related to long-term MCSD implantation are described in Chart 8.

**Chart 8:** Complications of long-term mechanical circulatory support devices (MCSDs)

Bleeding	Pericardial effusion	Respiratory insufficiency
Right ventricular dysfunction	Hypertension	Non-neurological arterial thromboembolism
Neurological events	Arrhythmias	Venous thromboembolism
Infections	Myocardial infarction	Surgical wound dehiscence
MCSD malfunction	Hepatic dysfunction	Psychiatric / behavioral change
Hemolysis	Renal dysfunction	

#### Proposal of prioritization criteria for cardiac transplant in patients with MCSD

With increasing number of MCDSs, this document proposes a change in the prioritization criteria for patients in the cardiac transplant waiting list. These new criteria are described in Chart 9.

**Chart 9:** Proposal of prioritization criteria for cardiac transplant

Priority	Criterium
1	Cardiogenic shock in patients using short/medium-term paracorporeal MCDS (including intra-aortic balloon) Long-term MCDS with complications and substitution of device is not possible
2	Cardiogenic shock in patients using inotropes or vasopressors
3	Stable long-term MCDS without complications
4	Outpatient management of advanced heart failure

MCDS: mechanical circulatory device support
